# Analysis of Worldwide Carrier Frequency and Predicted Genetic Prevalence of Autosomal Recessive Congenital Hypothyroidism Based on a General Population Database

**DOI:** 10.3390/genes12060863

**Published:** 2021-06-04

**Authors:** Kyung-Sun Park

**Affiliations:** Department of Laboratory Medicine, Kyung Hee University School of Medicine and Kyung Hee University Medical Center, Seoul 02447, Korea; drparkkyungsun@khu.ac.kr; Tel.: +82-2-958-8674

**Keywords:** congenital hypothyroidism, population database, carrier frequency, genetic prevalence, gnomAD database

## Abstract

To assess how genomic information of the general population reflects probabilities of developing diseases and the differences in those probabilities among ethnic groups, a general population database was analyzed with an example of congenital hypothyroidism. Twelve candidate genes that follow an autosomal recessive inheritance pattern in congenital hypothyroidism (*SLC5A5*, *TPO*, *TG*, *IYD*, *DUOXA2*, *DUOX2*, *TSHR*, *SLC26A7, GLIS3, FOXE1*, *TSHB*, *TRHR*) in the gnomAD database (v2.1.1) were analyzed. The carrier frequency (CF) and predicted genetic prevalence (pGP) were estimated. The total CF in the overall population was 3.6%. *DUOX2* showed the highest CF (1.8%), followed by *TG* (0.46%), *TPO* (0.44%), *TSHR* (0.31%), *SLC26A7* (0.144%), *DUOXA2* (0.141%), *IYD* (0.08%), *SLC5A5* (0.06%), *TRHR* (0.059%), *GLIS3* (0.059%), *TSHB* (0.04%), and *FOXE1* (0%). The pGP in the overall population was 10.01 individuals per 100,000 births (1:9992). The highest pGP was in the East Asian population at 52.48 per 100,000 births (1:1905), followed by Finnish (35.96), Non-Finnish European (9.56), African/African American (4.0), Latino/Admixed American (3.89), South Asian (3.56), and Ashkenazi Jewish (1.81) groups. Comparing the pGP with the real incidence of congenital hypothyroidism, the pGP in East Asian populations was highly consistent with the real incidence.

## 1. Introduction

Genetic screening is a type of genetic testing that is designed to identify a specified population at a higher risk of having or developing a disease with the aim of prevention or early treatment [1]. Generally, genetic screening is performed as targeted testing for known hotspot variations. The development of next-generation sequencing (NGS) techniques has introduced a new genomic era by producing massive genomic data and reducing costs. Recently, the Genome Aggregation Database (gnomAD, https://gnomad.broadinstitute.org/, accessed on 31 March 2021) has been constructed as a very large database that contains genomic information of the general population worldwide [2,3]. Several companies have launched proactive genetic testing for generally healthy individuals without a personal or family history using NGS techniques for identifying particular genes or performing whole exome/genome sequencing that is not confined to hotspot variations.

At present, major questions are how genomic information of a generally healthy population reflects probabilities of developing diseases and the differences in those probabilities among population groups. Additional questions are the use of genetic testing to provide useful and crucial information and eventually prevent diseases in healthy individuals. To answer these questions, genomic data associated with congenital hypothyroidism, which is one of the major achievements of preventive medicine [4], were analyzed based on the general population database, and their carrier frequency and genetic prevalence were estimated by population.

## 2. Materials and Methods

### 2.1. Database Analysis

The gnomAD data (v2.1.1) were obtained from https://gnomad.broadinstitute.org/ (accessed on 31 March 2021). A total of 12 candidate genes that follow an autosomal recessive inheritance pattern in congenital hypothyroidism (*SLC5A5*, *TPO*, *TG*, *IYD*, *DUOXA2*, *DUOX2*, *TSHR*, *SLC26A7, GLIS3, FOXE1*, *TSHB*, *TRHR*) were analyzed. The gnomAD database (v2.1.1) contains genetic variants from 125,748 exomes and 15,708 genomes (total number = 141,456) and 7 populations including 12,487 African/African American (AFR), 17,720 Latino/Admixed American (AMR), 5185 Ashkenazi Jewish (ASJ), 9977 East Asian (EAS), 12,562 Finnish (FIN), 64,603 Non-Finnish European (NFE), and 15,308 South Asian (SAS).

### 2.2. Genetic Variant Classification

The GRCh37/hg19 genomic build was used for all position descriptions. All variants were described according to HGVS variant nomenclature standards (http://varnomen.hgvs.org/, accessed on 31 March 2021) [5] and analyzed based on the transcript selected by matched annotation from NCBI and EMBL-EBI (MANE) (https://www.ncbi.nlm.nih.gov/refseq/MANE/, accessed on 31 March 2021) using the Mutalyzer program (https://mutalyzer.nl/, accessed on 31 March 2021).

All genetic variants in the 12 candidate genes reported in the gnomAD database (v.2.1.1) were classified following the 2015 American College of Medical Genetics and Genomics (ACMG)/Association for Molecular Pathology (AMP) standards and guidelines [6] and Sequence Variant Interpretation (SVI) general recommendations by ClinGen (https://clinicalgenome.org/working-groups/sequence-variant-interpretation/, accessed on 1 April 2021). Loss-of-function variants of these candidate genes were presumed to be responsible for a congenital hypothyroidism mechanism. Therefore, a PVS1 (pathogenic criterion for predicted loss of function variants) decision tree was applied for PVS1 ACMG/AMP variant criteria [7]. If the genetic variants were not known pathogenic or likely pathogenic variants (PLPVs), the null variants (stop-gain, splice site disrupting, or frameshift variants) with flags of low-confidence predicted loss-of-function (pLoF) or pLof flag by loss-of-function transcript effect estimator (LOFTEE, https://github.com/konradjk/loftee, accessed on 31 March 2021) were filtered. For the PM2 code, the method used to determine the PM2 threshold was adopted by the ClinGen inborn errors of metabolism (IEM) working group [8]; the most frequent pathogenic variant in 12 candidate genes in gnomAD is c.2895_2898delGTTC (p.Phe966SerfsTer29) in *DUOX2*, which has a minor allele frequency of 0.0029 (allele frequency of heterozygous pathogenic variant in the global population in gnomAD); therefore, the PM2 (absence/rarity) threshold was set at an order of magnitude lower, an allele frequency of 0.0003. The PM3 (in trans criterion) code was applied following the recommendation of the SVI working group (https://clinicalgenome.org/working-groups/sequence-variant-interpretation/, accessed on 1 April 2021); each proband was awarded points value, and then the strength level for PM3 was determined. In addition, the PM3 was applied considering the direction of avoiding circular logic. For the PP1 (co-segregation) code, the evidence strength was determined following the specification of the ClinGen inborn errors of metabolism (IEM) working group [8]. The PP4 and PP5 codes were not applied in this study. For the prediction of variant pathogenicity (PP3), multiple in silico software such as REVEL (>0.75 for missense variants, https://sites.google.com/site/revelgenomics/downloads?authuser=0, accessed on 31 March 2021) [9,10], Mutation Taster (http://www.mutationtaster.org/, accessed on 31 March 2021) [11], PROVEAN [12] (for in-frame insertion or deletion variants, http://provean.jcvi.org/index.php, accessed on 31 March 2021), and spliceAI (for predicted impact on splicing, https://spliceailookup.broadinstitute.org/, accessed on 31 March 2021) [13] were used. In addition, for checking critical functional domains when applying the PVS1 decision tree [7] or for applying the PM1 code, Pfam (https://pfam.xfam.org/, accessed on 1 April 2021), InterPro (https://www.ebi.ac.uk/interpro/, accessed on 1 April 2021), and UniProt (https://www.uniprot.org/, accessed on 1 April 2021) were used.

### 2.3. Carrier Frequency (CF) and Predicted Genetic Prevalence Analysis (pGP)

For CF and pGP analysis, only heterozygous PLPV (not homozygous PLPV) was considered [14,15,16]. Therefore, the allele frequency of heterozygous PLPV (AF_V_) and CF_V_ for a variant V were calculated as follows:

(1) $$AF_{V}=\frac{allele\;count-2*homozygous\;count}{allele\;number}$$

(2) $$CF_{V}=\frac{AF_{V\;}*\;allele\;number\;}{Number\;of\;individuals}=2AF_{V}$$

where the allele count (number of variant alleles), allele number (number of genotyped alleles = 2∗number of individuals), and homozygous count (number of homozygous individuals) for a variant were provided by gnomAD.

For the CF and pGP in a gene level (CF_G_ and pGP_G_, respectively), two methods were applied. The first method (method 1) followed CF_G_ and pGP_G_ calculations, as previously described [15] as follows:

(3) $$CF_{G}=1-\prod_{k=1}^{n}\left(1-CF_{V}\right)$$

(4) $$pGP_{G}=\frac{\sum_{k=1}^{n}{\left(CF_{V}\right)}_{ik}{\left(CF_{V}\right)}_{ik}}{4}$$

## 3. Results

### 3.1. Presumed (Likely) Pathogenic Variants in 12 Candidate Genes

The presumed PLPVs in the 12 candidate genes are described in Tables S1–S11. A total of 610 variants were classified into PLPVs: 18 variants in *SLC5A5*, 79 variants in *TPO*, 163 variants in *TG*, 20 variants in *IYD*, 34 variants in *DUOXA2*, 143 variants in *DUOX2*, 53 variants in *TSHR*, 46 variants in *SLC26A7*, 28 variants in *GLIS3*, no variant in *FOXE1*, 8 variants in *TSHB*, and 18 variants in *TRHR* (Table 1).

These 610 variants included 218 nonsense variants (35.7%), 213 frameshift variants (34.9%), 125 splice variants (20.5%), 51 missense variants (8.4%), and 3 in-frame deletion variants (0.5%) (Tables S1–S11). Of the 610 variants, only 89 variants (14.6%) were registered in the ClinVar database (https://www.ncbi.nlm.nih.gov/clinvar/, accessed on 28 May 2021): 22.2% in *SLC5A5*, 19.0% in *TPO*, 9.8% in *TG*, 20.0% in *IYD*, 11.8% in *DUOXA2*, 17.5% in *DUOX2*, 24.5% in *TSHR*, 2.2% in *SLC26A7*, 3.6% in *GLIS3*, 50% in *TSHB*, and 11.1% in *TRHR*. The representative PLPVs with allele frequency greater than 0.0001 (in global population in gnomAD) in the 12 candidate genes were described in Table 2.

There were 4 variants in *TPO*, 3 variants in *TG*, 1 variant in *IYD*, 2 variants in *DUOXA2*, 10 variants in *DUOX2*, 4 variants in *TSHR*, 1 variant in *SLC26A7,* 1 variant in *TSHB*, and 1 variant in *TRHR.*

### 3.2. Distribution of Carrier Frequency and Predicted Genetic Prevalence in Each Population Group

**Figure 1:**
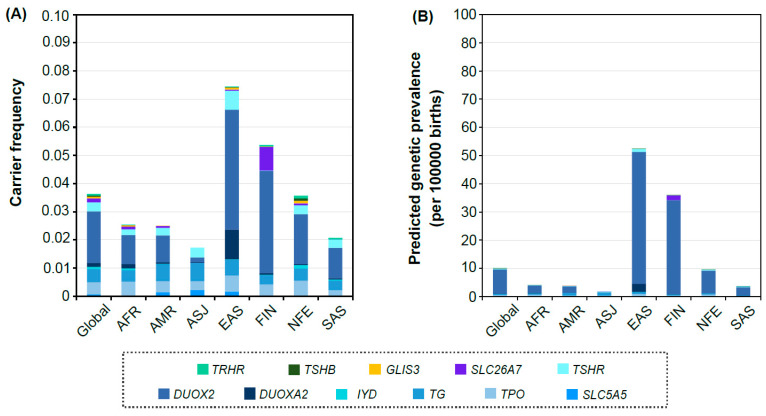
Carrier frequency and predicted genetic prevalence. (**A**) Distribution of carrier frequency in each population. (**B**) Distribution of predicted genetic prevalence in each population.

The total CF for all 12 candidate genes in the overall population was 3.6% (Figure 1A). This means that unaffected carriers among 12 candidate genes were predicted to be 3.6%. Among the seven population groups, the EAS population showed the highest total CF (7.4%), followed by FIN (5.4%), NFE (3.6%), AFR (2.54%), AMR (2.51%), SAS (2.1%), and ASJ (1.7%). In the ASJ population, any presumed PLPV in *IYD*, SLC26A7, *GLIS3, FOXE1*, *TSHB*, and *TRHR* were not found. In the global population, of the 12 candidate genes, *DUOX2* showed the highest CF (1.8%), followed by *TG* (0.46%), *TPO* (0.44%), *TSHR* (0.31%), *SLC26A7* (0.144%), *DUOXA2* (0.141%), *IYD* (0.08%), *SLC5A5* (0.06%), *TRHR* (0.059%), *GLIS3* (0.059%), *TSHB* (0.04%), and *FOXE1* (0%). Interestingly, the distribution of the proportion of genes in the EAS population was unique: *DUOX2* (4.25%) > *DUOXA2* (1.06%) > *TSHR* (0.67%) > *TG* (0.57%) > *TPO* (0.57%) > *SLC5A5* (0.16%) > *GLIS3* (0.08%) > *SLC26A7* (0.04%) > *TRHR* (0.02%) > *TSHB* (0.01%) > *IYD* (0.01%) > *FOXE1* (0%).

Overall, the pGP caused by 12 candidate genes in the total population was 10.01 individuals per 100,000 births (1:9992) (Figure 1B). The pGP of the EAS population was 52.48 per 100,000 births (1:1905), followed by FIN (35.96 per 100,000 births, 1:2781), NFE (9.56 per 100,000 births, 1:10,457), AFR (4.0 per 100,000 births, 1:24,981), AMR (3.89 per 100,000 births, 1:25,728), SAS (3.56 per 100,000 births, 1:28,103), and ASJ (1.81 per 100,000 births, 1:55,282).

## 4. Discussion

Congenital hypothyroidism is the most common neonatal disorder [17]. Prompt diagnosis and treatment may help prevent patient intellectual disability [18]. The newborn screening program for congenital hypothyroidism with detection of blood spot thyroid stimulating hormone (TSH) or thyroxine (T4) was implemented between 1970 and 1980 worldwide, especially in developed countries. This public health program has nearly eradicated the profound physical and cognitive impairments due to severe congenital hypothyroidism. Recent studies raised the issue that current screening criteria miss borderline or subclinical congenital hypothyroidism [19,20].

Primary congenital hypothyroidism is broadly caused by thyroid dysgenesis (including agenesis, hypoplasia, or abnormal location) or dyshormogenesis (when a normal thyroid gland produces abnormal amounts of thyroid hormone). Historically, the most common cause (approximately 85%) of primary hypothyroidism is thyroid dysgenesis [18,21,22,23], with an incidence of about 1:4000 births. However, thyroid dysgenesis occurs sporadically, and fewer than 5% of thyroid dysgenesis cases are attributable to genetic variations in the known genes. Dyshormogenesis accounts for approximately 15% of primary hypothyroidism and is mainly caused by a genetic defect. The proportion of dyshormogenesis cases within congenital hypothyroidism has been increasing up to over 30% [18,21].

Genes associated with congenital hypothyroidism are the following: genes associated with thyroid dysgenesis or syndromic primary congenital hypothyroidism (for simplicity, thyroid dysgenesis, *NKX2-1*, *FOXE1*, *PAX8*, *NKX2-5*, *GLIS3*, *JAG1*, *TBX1*, *NTN1*, *CDCA8*, *TUBB1*), genes associated with thyroid dyshormonogenesis (*TSHR*, *GNAS*, *SLC5A5*, *SLC26A4*, *TPO*, *TG*, *IYD*, *DUOXA2*, *DUOX1*, *DUOX2*, *SLC26A7*), genes associated with isolated central congenital hypothyroidism (*TSHB*, *TRHR*, *TBL1X*, *IRS4*), genes associated with multiple pituitary hormone deficiencies (*IGSF1*, *PROP1*, *POU1F1*, *HESX1*, *SOX3*, *OTX2*, *LHX3*, *LHX4*, *LEPR*, *SOX2*), and genes associated with other central congenital hypothyroidism (*PROKR2*, *NFKB2*, *CHD7*, *FGFR1*, *FGF8*, *FOXA2*) [24]. Especially, most thyroid dyshormonogenesis genes are inherited in an autosomal recessive pattern, while most thyroid dysgenesis and their related conditions are autosomal dominant [24,25]. In this study, the carrier frequency and predicted genetic prevalence of autosomal recessive congenital hypothyroidism were analyzed based on the general population database. Most of the general population is regarded to include individuals without severe hypothyroid conditions; therefore, only genes inherited in an autosomal recessive pattern were included in this study. Therefore, this study analyzed 12 genes associated with autosomal recessive thyroid dysgenesis, thyroid dyshormonogenesis, or isolated central congenital hypothyroidism based on gnomAD (v2.1.1).

Differences in the prevalence of congenital hypothyroidism by population have been reported [17,26,27] (Table S12). The Asian and Latino (Hispanic) groups showed higher rates, while the African population had a lower rate compared with the incidence of congenital hypothyroidism in the European group. In this study, the pGP of congenital hypothyroidism in EAS (1:1905) was notably higher than other populations and consistent with the incidence based on newborn screening programs in EAS (1:1443–1:2380) (Table S12). However, in contrast to the previous studies (1:1404–1:4149, Table S12), the AMR in this study (1:25,728) showed the lower rate of pGP for congenital hypothyroidism. In addition, there was a difference between the pGP and real incidence of congenital hypothyroidism in other populations except the EAS group.

The difference between the pGP based on the population database and the real incidence might be determined by how many genes following autosomal recessive inheritance patterns were associated with their diseases by population group, because the pGP in this study was calculated not considering autosomal dominant or X-linked inheritance; in a larger proportion of genes that follow an autosomal recessive inheritance pattern within the entire genetic portion, the gap between the pGP and real prevalence is narrowing. There are differences between the proportions of thyroid dysgenesis and dyshormonogenesis between ethnic groups [26]. In particular, the proportion of dyshormonogenesis in congenital hypothyroidism in Asian is higher than that in Caucasian [26]. Since most pathogenic variations associated with dyshormonogenesis are inherited in an autosomal recessive manner, if the proportion of thyroid dyshormonogenesis is higher in the specific population, the pGP would be more consistent with the real incidence. These results may indicate why the pGP of the EAS group in this study was more consistent with the real incidence. Recent studies using NGS have reported that among the causes of congenital hypothyroidism in East Asians, thyroid dyshormonogenesis is higher than thyroid dysgenesis or others [28,29,30]. In contrast, if the proportion of dyshormonogenesis in a population is lower, the difference between pGP and real incidence would be bigger because the genetic cause from thyroid dysgenesis would be underestimated; many of the thyroid dysgenesis genes are inherited in an autosomal dominant manner.

Another important reason for the difference between the pGP and real incidence is that this study simplified the pGP by gene unit and assumed that congenital hypothyroidism occurs only when two (likely) pathogenic variants are present in one gene, depending on the inheritance of autosomal recessivity. However, a congenital hypothyroidism has the genetic heterogeneity. Not all the genetic factors associated with congenital hypothyroidism have yet been identified. Recently, it has been identified that loss-of-function variants in *SLC26A7* are another genetic cause of dyshormonogenesis [31]. When testing already known genes in cohorts of patients with congenital hypothyroidism using NGS, only one causative variant is often identified in genes related to autosomal recessive inheritance [32,33,34,35]. This suggests that no other causative variant has been found due to limitations in genetic testing (e.g., variant in deep intron regions) or interpretation for genetic variants (e.g., variants of uncertain significance, VUS), or that the genetic variant found in patients has been inherited in a different pattern (e.g., dominant negative). In addition, a congenital hypothyroidism might have a digenic cause; digenic *DUOX1* and *DUOX2* causative variants in cases with congenital hypothyroidism have been reported [36].

Generally, if the specific variant is submitted to ClinVar as PLPV, it means that clinical patients with those PLPVs are present, and those PLPVs are the main cause of their disease development. In this study, the results showed that only 14.5% variants were submitted to ClinVar. In addition, genetic studies on congenital hypothyroidism have been analyzed based on specific populations. Therefore, the CF and pGP might be underestimated in the population that showed the biggest difference between the pGP and real prevalence, because many variants would be classified as VUS and not as PLPVs due to insufficiency of genetic and clinical information. Especially with the application of ClinGen recommendations, even if the variants were detected repeatedly in the patients, the evidence with higher weights cannot be applied without functional studies or family analysis of the variants. For the classification of variants associated with congenital hypothyroidism, establishment of the threshold weight of each functional study with respect to the PS3 code is needed. Additionally, epidemiologic or environmental factors [27,37] also are attributable to the difference between pGP and prevalence.

## 5. Conclusions

In conclusion, this is the first study that assessed congenital hypothyroidism based on general population data and estimated CF and pGP by population. In particular, comparing the pGP with the real incidence of congenital hypothyroidism, the pGP in East Asian populations was highly consistent with the real incidence. The approach to obtain genomic information of a general population would allow an additional and helpful direction for preventive medicine. However, when using genomic information from the general population, the pathogenesis of particular diseases should be considered by population group.

## Figures and Tables

**Table 1:** Presumed (likely) pathogenic variants in 12 genes associated with autosomal recessive congenital hypothyroid found in gnomAD.

Group	Gene	No. of Presumed Pathogenic or Likely Pathogenic Variants							
		Global	AFR	AMR	ASJ	EAS	FIN	NFE	SAS
DH	*SLC5A5*	18	1	3	2	5	8	6	3
	*TPO*	79	15	13	4	9	3	51	9
	*TG*	163	25	29	5	25	5	79	32
	*IYD*	20	5	1	0	1	1	14	3
	*DUOXA2*	34	8	4	1	4	1	12	4
	*DUOX2*	143	28	37	3	39	6	74	24
	*TSHR*	53	14	11	1	5	2	33	6
	*SLC26A7*	46	8	8	1	6	1	27	5
TD	*GLIS3*	28	5	3	0	4	3	18	0
	*FOXE1*	0	0	0	0	0	0	0	0
ICCH	*TSHB*	8	1	0	0	1	1	6	1
	*TRHR*	18	1	0	0	1	4	15	1

DH, thyroid dyshormonogenesis; TD, thyroid dysgenesis or syndromic primary congenital hypothyroidism; ICCH, isolated central congenital hypothyroidism; AFR, African/African American; AMR, Latino/Admixed American; ASJ, Ashkenazi Jewish; EAS, East Asian; FIN, Finnish; NFE, Non-Finnish European; SAS, South Asian.

**Table 2:** Representative pathogenic or likely pathogenic variants with allele frequency greater than 0.0001 in global population in gnomAD.

Genes ^1^	Variant	Allele frequency in gnomAD (v2.1.1)								
		Major Population ^2^	Global	AFR	AMR	ASJ	EAS	FIN	NFE	SAS
*TPO*	c.483-1G>C	AFR	0.00013	0.00133	0.00004	0	0	0	0.00001	0
	c.1184_1187dupGCCG, (p.Ala397ProfsTer76)	FIN, NFE, AMR	0.00053	0	0.00037	0	0	0.00156	0.00092	0
	c.1978C>G, (p.Gln660Glu)	AMR, NFE	0.00030	0.00008	0.00119	0	0	0	0.00023	0
	c.2268dupT, (p.Glu757Ter)	EAS	0.00012	0	0	0	0.00165	0	0	0
*TG*	c.886C>T, (p.Arg296Ter)	ASJ, AMR, NFE, AFR	0.00035	0.00028	0.00059	0.00106	0	0.00004	0.00042	0.00003
	c.1963C>T, (p.Gln655Ter)	FIN	0.00013	0	0	0	0	0.00140	0.00002	0
	c.5184C>A, (p.Cys1728Ter)	ASJ	0.00010	0	0.00003	0.00188	0	0	0.00003	0
*IYD*	c.315_317delCAT, (p.Phe105_Ile106delinsLeu)	NFE	0.00011	0.00008	0	0	0	0	0.00022	0
*DUOXA2*	c.413dupA, (p.Tyr138Ter)	EAS	0.00023	0	0	0	0.00333	0	0	0
	c.738C>G, (p.Tyr246Ter)	EAS	0.00014	0	0	0	0.00186	0	0.00001	0
*DUOX2*	c.4524+1dupG	AFR	0.00011	0.00126	0.00003	0	0	0	0	0
	c.3693+1G>T	EAS	0.00011	0	0	0	0.00160	0	0	0
	c.3329G>A, (p.Arg1110Gln)	EAS	0.00019	0	0.00003	0	0.00251	0	0.00002	0
	c.3155G>A, (p.Cys1052Tyr)	FIN, NFE, AMR, AFR	0.00128	0.00012	0.00014	0.00010	0	0.00629	0.00140	0
	c.2895_2898delGTTC, (p.Phe966SerfsTer29)	FIN, NFE, SAS, AMR, AFR	0.00290	0.00056	0.00158	0	0	0.01139	0.00290	0.00213
	c.1588A>T, (p.Lys530Ter)	EAS	0.00063	0	0	0	0.00882	0	0	0
	c.1516G>A, (p.Asp506Asn)	NFE, FIN	0.00026	0.00004	0.00003	0	0	0.00040	0.00047	0
	c.1462G>A, (p.Gly488Arg)	EAS, AFR	0.00015	0.00052	0	0	0.00120	0	0.00004	0
	c.1060C>T, (p.Arg354Trp)	AMR, SAS, NFE	0.00016	0.00008	0.00037	0	0.00005	0	0.00012	0.00029
	c.602dupG, (p.Gln202ThrfsTer99)	NFE, SAS, AMR, AFR, FIN	0.00086	0.00032	0.00055	0	0	0.00012	0.00158	0.00070
*TSHR*	c.202C>T, (p.Pro68Ser)	ASJ, SAS, AMR, NFE	0.00049	0.00004	0.00068	0.00174	0	0	0.00043	0.00115
	c.484C>G, (p.Pro162Ala)	NFE, EAS	0.00014	0.00004	0.00008	0	0.00010	0	0.00025	0
	c.1349G>A, (p.Arg450His)	EAS, SAS	0.00021	0	0.00008	0	0.00241	0.00004	0.00003	0.00013
	c.1637G>A, (p.Trp546Ter)	NFE	0.00011	0.00008	0	0	0	0	0.00022	0
*SLC26A7*	c.1893delT, (p.Phe631LeufsTer8)	FIN, NFE	0.00045	0.00004	0.00003	0	0	0.00420	0.00010	0
*TSHB*	c.373delT, (p.Cys125ValfsTer10)	NFE	0.00016	0.00004	0	0	0	0.00004	0.00032	0
*TRHR*	c.1016delA, (p.Gln339ArgfsTer14)	NFE	0.00012	0.00004	0	0	0	0	0.00024	0

^1^ All genes are inherited in an autosomal recessive pattern. *TPO, TG, IYD, DUOXA2, DUOX2, TSHR*, and *SLC26A7* genes are associated with thyroid dyshormonogenesis. *TSHB* and *TRHR* genes are associated with isolated central congenital hypothyroidism. ^2^ Order of high frequency (greater than allele frequency 0.0001) in population. AFR, African/African American; AMR, Latino/Admixed American; ASJ, Ashekenazi Jewish; EAS, East Asian; FIN, Finnish; NFE, Non-Finnish European; SAS, South Asian.

## Data Availability

All data analyzed in this study are included in this article and its supplementary information files.

## Supplementary Materials

The following are available online at https://www.mdpi.com/article/10.3390/genes12060863/s1, Table S1: Pathogenic or likely pathogenic variants in *SLC5A5* gene, Table S2: Pathogenic or likely pathogenic variants in *TPO* gene, Table S3: Pathogenic or likely pathogenic variants in *TG* gene, Table S4: Pathogenic or likely pathogenic variants in *IYD* gene, Table S5: Pathogenic or likely pathogenic variants in *DUOXA2* gene, Table S6: Pathogenic or likely pathogenic variants in *DUOX2* gene, Table S7: Pathogenic or likely pathogenic variants in *TSHR* gene, Table S8: Pathogenic or likely pathogenic variants in *TSHB* gene, Table S9: Pathogenic or likely pathogenic variants in *TRHR* gene, Table S10: Pathogenic or likely pathogenic variants in *SLC26A7* gene, Table S11: Pathogenic or likely pathogenic variants in *GLIS3* gene, Table S12: Incidence of congenital hypothyroidism.

## References

[1] Andermann A., Blancquaert I. (2010). Genetic screening: A primer for primary care. Can. Fam. Physician.

[2] Karczewski K.J., Francioli L.C., Tiao G., Cummings B.B., Alfoldi J., Wang Q., Collins R.L., Laricchia K.M., Ganna A., Birnbaum D.P. (2020). The mutational constraint spectrum quantified from variation in 141,456 humans. Nature.

[3] Lek M., Karczewski K.J., Minikel E.V., Samocha K.E., Banks E., Fennell T., O’Donnell-Luria A.H., Ware J.S., Hill A.J., Cummings B.B. (2016). Analysis of protein-coding genetic variation in 60,706 humans. Nature.

[4] Büyükgebiz A. (2013). Newborn Screening for Congenital Hypothyroidism. J. Clin. Res. Pediatr. Endocrinol..

[5] Dunnen J.T.D., Dalgleish R., Maglott D.R., Hart R.K., Greenblatt M.S., McGowan-Jordan J., Roux A.-F., Smith T., Antonarakis S.E., Taschner P.E. (2016). HGVS Recommendations for the Description of Sequence Variants: 2016 Update. Hum. Mutat..

[6] Richards S., Aziz N., Bale S., Bick D., Das S., Gastier-Foster J., Grody W.W., Hegde M., Lyon E., Spector E. (2015). Standards and guidelines for the interpretation of sequence variants: A joint consensus recommendation of the American College of Medical Genetics and Genomics and the Association for Molecular Pathology. Genet. Med..

[7] Tayoun A.N.A., Pesaran T., DiStefano M.T., Oza A., Rehm H.L., Biesecker L.G., Harrison S.M., ClinGen Sequence Variant Interpretation Working Group (ClinGen SVI) (2018). Recommendations for interpreting the loss of function PVS1 ACMG/AMP variant criterion. Hum. Mutat..

[8] Zastrow D.B., Baudet H., Shen W., Thomas A., Si Y., Weaver M.A., Lager A.M., Liu J., Mangels R., Dwight S.S. (2018). Unique aspects of sequence variant interpretation for inborn errors of metabolism (IEM): The ClinGen IEM Working Group and the Phenylalanine Hydroxylase Gene. Hum. Mutat..

[9] Ioannidis N.M., Rothstein J.H., Pejaver V., Middha S., McDonnell S.K., Baheti S., Musolf A., Li Q., Holzinger E., Karyadi D. (2016). REVEL: An Ensemble Method for Predicting the Pathogenicity of Rare Missense Variants. Am. J. Hum. Genet..

[10] Ghosh R., Oak N., Plon S.E. (2017). Evaluation of in silico algorithms for use with ACMG/AMP clinical variant interpretation guidelines. Genome Biol..

[11] Schwarz J.M., Cooper D.N., Schuelke M., Seelow D. (2014). MutationTaster2: Mutation prediction for the deep-sequencing age. Nat. Methods.

[12] Choi Y., Chan A.P. (2015). PROVEAN web server: A tool to predict the functional effect of amino acid substitutions and indels. Bioinformatics.

[13] Jaganathan K., Panagiotopoulou S.K., McRae J.F., Darbandi S.F., Knowles D., Li Y.I., Kosmicki J.A., Arbelaez J., Cui W., Schwartz G.B. (2019). Predicting Splicing from Primary Sequence with Deep Learning. Cell.

[14] Hanany M., Allon G., Kimchi A., Blumenfeld A., Newman H., Pras E., Wormser O., Birk O.S., Gradstein L., Banin E. (2018). Carrier frequency analysis of mutations causing autosomal-recessive-inherited retinal diseases in the Israeli population. Eur. J. Hum. Genet..

[15] Hanany M., Rivolta C., Sharon D. (2020). Worldwide carrier frequency and genetic prevalence of autosomal recessive inherited retinal diseases. Proc. Natl. Acad. Sci. USA.

[16] Park K.S. (2021). Carrier frequency and predicted genetic prevalence of Pompe disease based on a general population database. Mol. Genet. Metab. Rep..

[17] Feuchtbaum L., Carter J., Dowray S., Currier R.J., Lorey F. (2012). Birth prevalence of disorders detectable through newborn screening by race/ethnicity. Genet. Med..

[18] Cherella C.E., Wassner A.J. (2017). Congenital hypothyroidism: Insights into pathogenesis and treatment. Int. J. Pediatr. Endocrinol..

[19] Leonardi D., Polizzotti N., Carta A., Gelsomino R., Sava L., Vigneri R., Calaciura F. (2008). Longitudinal Study of Thyroid Function in Children with Mild Hyperthyrotropinemia at Neonatal Screening for Congenital Hypothyroidism. J. Clin. Endocrinol. Metab..

[20] Lain S.J., Bentley J.P., Wiley V., Roberts C.L., Jack M., Wilcken B., Nassar N. (2016). Association between borderline neonatal thyroid-stimulating hormone concentrations and educational and developmental outcomes: A population-based record-linkage study. Lancet Diabetes Endocrinol..

[21] Wassner A.J., Brown R.S. (2015). Congenital hypothyroidism: Recent advances. Curr. Opin. Endocrinol. Diabetes Obes..

[22] Olivieri A., Fazzini C., Medda E. (2015). The Italian Study Group for Congenital Hypothyroidism Multiple Factors Influencing the Incidence of Congenital Hypothyroidism Detected by Neonatal Screening. Horm. Res. Paediatr..

[23] Deladoëy J., Ruel J., Giguère Y., van Vliet G. (2011). Is the Incidence of Congenital Hypothyroidism Really Increasing? A 20-Year Retrospective Population-Based Study in Québec. J. Clin. Endocrinol. Metab..

[24] Van Trotsenburg P., Stoupa A., Léger J., Rohrer T., Peters C., Fugazzola L., Cassio A., Heinrichs C., Beauloye V., Pohlenz J. (2021). Congenital Hypothyroidism: A 2020–2021 Consensus Guidelines Update—An ENDO-European Reference Network Initiative Endorsed by the European Society for Pediatric Endocrinology and the European Society for Endocrinology. Thyroid.

[25] Peters C., van Trotsenburg A.S.P., Schoenmakers N. (2018). Diagnosis of Endocrine Disease: Congenital hypothyroidism: Update and perspectives. Eur. J. Endocrinol..

[26] Stoppa-Vaucher S., van Vliet G., Deladoëy J. (2011). Variation by Ethnicity in the Prevalence of Congenital Hypothyroidism Due to Thyroid Dysgenesis. Thyroid.

[27] Hinton C.F., Harris K.B., Borgfeld L., Drummond-Borg M., Eaton R., Lorey F., Therrell B.L., Wallace J., Pass K.A. (2010). Trends in Incidence Rates of Congenital Hypothyroidism Related to Select Demographic Factors: Data from the United States, California, Massachusetts, New York, and Texas. Pediatrics.

[28] Sun F., Zhang J.-X., Yang C.-Y., Gao G.-Q., Zhu W.-B., Han B., Zhang L.-L., Wan Y.-Y., Ye X.-P., Ma Y.-R. (2018). The genetic characteristics of congenital hypothyroidism in China by comprehensive screening of 21 candidate genes. Eur. J. Endocrinol..

[29] Park K.-J., Park H.-K., Kim Y.-J., Lee K.-R., Park J.-H., Park J.-H., Park H.-D., Lee S.-Y., Kim J.-W. (2016). DUOX2 Mutations Are Frequently Associated with Congenital Hypothyroidism in the Korean Population. Ann. Lab. Med..

[30] Yu B., Long W., Yang Y., Wang Y., Jiang L., Cai Z., Wang H. (2018). Newborn Screening and Molecular Profile of Congenital Hypothyroidism in a Chinese Population. Front. Genet..

[31] Cangul H., Liao X.-H., Schoenmakers E., Kero J., Barone S., Srichomkwun P., Iwayama H., Serra E.G., Saglam H., Eren E. (2018). Homozygous loss-of-function mutations in SLC26A7 cause goitrous congenital hypothyroidism. JCI Insight.

[32] Stoupa A., Chehade G.A.H., Chaabane R., Kariyawasam D., Szinnai G., Hanein S., Bole-Feysot C., Fourrage C., Nitschke P., Thalassinos C. (2020). High Diagnostic Yield of Targeted Next-Generation Sequencing in a Cohort of Patients with Congenital Hypothyroidism Due to Dyshormonogenesis. Front. Endocrinol..

[33] Shin J.H., Kim H.Y., Kim Y.M., Lee H., Bae M.H., Park K.H., Lee S.-M., Kwak M.J. (2021). Genetic Evaluation of Congenital Hypothyroidism with Gland in situ Using Targeted Exome Sequencing. Ann. Clin. Lab. Sci..

[34] Makretskaya N., Bezlepkina O., Kolodkina A., Kiyaev A., Vasilyev E.V., Petrov V., Kalinenkova S., Malievsky O., Dedov I.I., Tiulpakov A. (2018). High frequency of mutations in ’dyshormonogenesis genes’ in severe congenital hypothyroidism. PLoS ONE.

[35] Long W., Lu G., Zhou W., Yang Y., Zhang B., Zhou H., Jiang L., Yu B. (2018). Targeted next-generation sequencing of thirteen causative genes in Chinese patients with congenital hypothyroidism. Endocr. J..

[36] Aycan Z., Cangul H., Muzza M., Bas V.N., Fugazzola L., Chatterjee V.K., Persani L., Schoenmakers N. (2017). Digenic DUOX1 and DUOX2 Mutations in Cases with Congenital Hypothyroidism. J. Clin. Endocrinol. Metab..

[37] Medda E., Olivieri A., Stazi M.A., Grandolfo M.E., Fazzini C., Baserga M., Burroni M., Cacciari E., Calaciura F., Cassio A. (2005). Risk factors for congenital hypothyroidism: Results of a population case-control study (1997–2003). Eur. J. Endocrinol..

